# The International Vertebrate Pet Trade Network and Insights from US Imports of Exotic Pets

**DOI:** 10.1093/biosci/biab056

**Published:** 2021-06-09

**Authors:** James S Sinclair, Oliver C Stringham, Bradley Udell, Nicholas E Mandrak, Brian Leung, Christina M Romagosa, Julie L Lockwood

**Affiliations:** Aquatic Ecology Lab, The Ohio State University, Columbus, Ohio, United States; Invasion Science and Wildlife Ecology Lab, The University of Adelaide, Adelaide, South Australia, Australia; University of Florida, Gainesville, and is currently an ecologist with the US Geological Survey's North American Bat Monitoring Program, Fort Collins Science Center, Fort Collins, Colorado, United States; University of Toronto Scarborough, Toronto, Ontario, Canada; McGill University, UNESCO Chair for Dialogues on Sustainability, and the Director of the McGill-STRI Neotropical Environment Option, Montreal, Quebec, Canada; University of Florida, Gainesville, Florida, United States; Department of Ecology, Evolution, and Natural Resources, Rutgers University, New Brunswick, New Jersey, United States

## Abstract

The international trade in exotic vertebrate pets provides key social and economic benefits but also drives associated ecological, ethical, and human health impacts. However, despite its clear importance, we currently lack a full understanding of the structure of the pet trade, hampering efforts to optimize its benefits while mitigating its negative effects. In the present article, we represent and review the structure of the pet trade as a network composed of different market actors (nodes) and trade flows (links). We identify key data gaps in this network that, if filled, would enable network analyses to pinpoint targets for management. As a case study of how data-informed networks can realize this goal, we quantified spatial and temporal patterns in pets imported to the United States. Our framework and case study illustrate how network approaches can help to inform and manage the effects of the growing demand for exotic pets.

The legal, international transport of undomesticated vertebrates as pets (hereafter, *pet trade*) is widespread and growing (Bush et al. 2014, Lockwood et al. 2019), involving billions of dollars in trade and millions of animals each year (Karesh et al. 2007, Bush et al. 2014). The pet trade is therefore a key economic resource, particularly for local traders that depend on it for their income (e.g., Raghavan et al. 2013, Robinson et al. 2018). Pets also provide important social and human health benefits (McNicholas et al. 2005), even being considered as family members within some cultures (Lockwood et al. 2019). However, the pet trade is also responsible for the widespread translocation of biota and associated pathogens across the globe, which can have a variety of negative impacts, including species harvested to near extinction (Nijman et al. 2012, Poole and Shepherd 2017), animal abuse (Baker et al. 2013, Warwick 2014, Elwin et al. 2020), biological invasions (Kraus 2008, Lockwood et al. 2019), and the introduction of new zoonotic diseases that harm native species and human health (Chomel et al. 2007, Smith et al. 2017, O'Hanlon et al. 2018). Retaining the positive benefits of the pet trade while addressing its associated negative impacts therefore requires an understanding of the full structure of, and flow of animals through, the pet trade commodity chain.

Despite the broad scope and clear importance of the pet trade on a variety of economic, cultural, ecological, ethical, and human health systems, a comprehensive understanding and approach to mitigating the negative effects of this trade remains elusive (Rhyne et al. 2017, Smith et al. 2017, Scheffers et al. 2019). This implementation gap persists because, outside of select taxonomic groups, there is scarce empirical information on where wild animals are initially harvested to supply the pet market or the precise volume traded between regions. We also lack critical information on the full spectrum of species traded in these markets, the dynamics of traded species over space and time, and how imported pets are geographically distributed in the countries into which they are sold. Data that are readily available often come from wildlife export and import records (e.g., Rhyne et al. 2012, Bush et al. 2014, Reino et al. 2017), which frequently address trade only at the coarse scale of whole countries, are not specific to the pet trade (e.g., include food and product animals), or misidentify the species involved (Rhyne et al. 2012). Furthermore, available pet trade data that are specific to finer-scale activities are typically highly species specific (e.g., Rabemananjara et al. 2008, Nijman et al. 2012, Martin et al. 2018). Taken together, the limited empirical understanding of the pet trade hampers our ability to extrapolate to the broader trade commodity chain, which consists of a variety of species traded across a complex web of actors spanning multiple spatial and temporal scales. Without this more comprehensive view of the pet trade, the suite of policy and enforcement solutions that can mitigate its negative effects remain opaque and therefore difficult to realize in practice.

## The pet trade is a spatial network

We posit that addressing empirical data gaps across the global pet trade and mitigating its negative effects can be better accomplished by representing the trade as a spatially explicit network, with each of the actors and their trade interactions treated as respective nodes and links in a network model (Reino et al. 2017, Vall-llosera and Cassey 2017, Frost et al. 2019). In this trade network, pets originate in a source region and are transported from one linked node to the next along the commodity chain until their eventual point of retail sale and ultimate use. Actors in the trade serve as the nodes because they have a physical location in which pets are received, stored, and transported through the network. This approach provides a suitable framework for the pet trade by summarizing the principal market actors involved (the nodes), helping to quantify the spatial patterns and trade flows of pets (the links), standardizing terminology, and targeting data gathering efforts to key network components. Well-known network-level and node-level statistics can also be used to identify trade hubs to prioritize for information gathering or management intervention, and to identify trading partners that are highly influential in structuring the trade network (Reino et al. 2017, Vall-llosera and Cassey 2017, Sánchez*‐*Mercado et al. 2020). Such network approaches are powerful tools to map the topology of the pet trade and to focus management to the areas that best minimize its negative impacts (box 1).

**Box 1. Insights from pet trade networks.**

Pre- and postimport networks can be used to improve estimates of source harvest and the risks associated with pet introduction, which we illustrate using a hypothetical and simplified example of a trade network (right figure). In this example, pets are initially collected across a network of geographically dispersed harvesting locations (green nodes) and consolidated at an export location (purple node). Following arrival at the port of entry (pink node), pets are then redistributed into successively more geographically dispersed areas (orange then red nodes). On the basis of the nodes and link strengths (represented using line thickness) in this network, we can determine that the greatest harvest intensity occurs in the topmost green node, that the purple and pink nodes act as key transport hubs, and that most pets are eventually sold in the topmost red node, which may therefore be at higher risk of disease transmission or invasion.
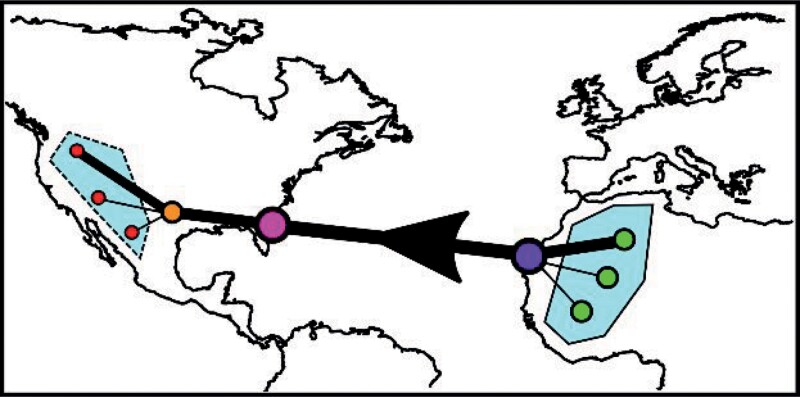

Spatial information on the pet network can also be used to estimate disease or invasion risk through environmental matching. Using another simplified example network (see the left figure), animals for the trade may be primarily harvested in one environment (blue) but sold in a different environment (yellow), creating an environmental mismatch. Therefore, successful disease transmission or nonnative species establishment may be less likely because pets are primarily sold into an unsuitable or unfamiliar environment. Conversely, the smaller trade routes between similar environments (i.e., blue to blue or yellow to yellow) or secondary transport from an unsuitable to a suitable region (e.g., blue region imports transported across the red dashed line) may be more important targets for management efforts.
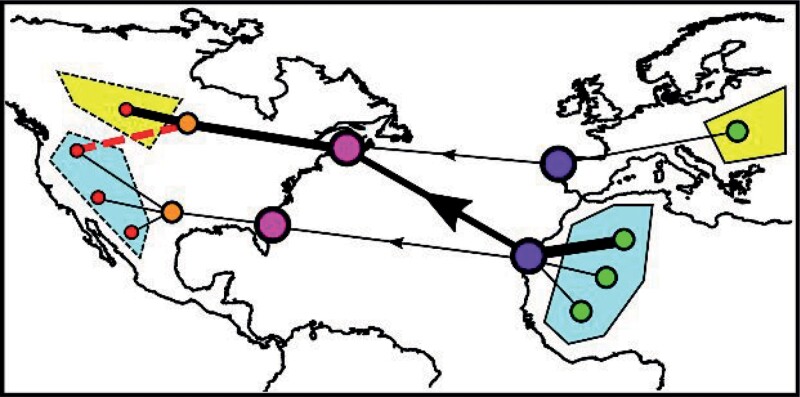

To develop the network structure of the pet trade, we first systematically gathered published literature on international pet trade commodity chains to build a comprehensive, composite representation of the different types of actors and trade connections (see supplemental file S1 for the methodology). Using this composite representation as an information framework, we then conducted a second review designed to evaluate gaps in empirical data related to each type of node and link (see supplemental file S1). Finally, we used a case study of vertebrates imported to the continental United States to illustrate the value of a network approach. The United States is a principal driver of the global pet trade (Kraus 2008, Bush et al. 2014, Ribeiro et al. 2019) and our analyses show how some of the best available data on this trade fits into our framework and how a better understanding of spatial and temporal trade patterns can reveal key targets for management efforts. We also use this case study to infer potential spatial patterns of where traded individuals were likely harvested and where they are imported within the United States. These initial inflows and final outflows of the trade network are poorly understood but are crucial for evaluating the extinction risk of vertebrates feeding the pet trade and its role in introducing nonnative animals and their associated pathogens.

## The pet trade commodity chain is a complex, multistage process

**Figure 1.**
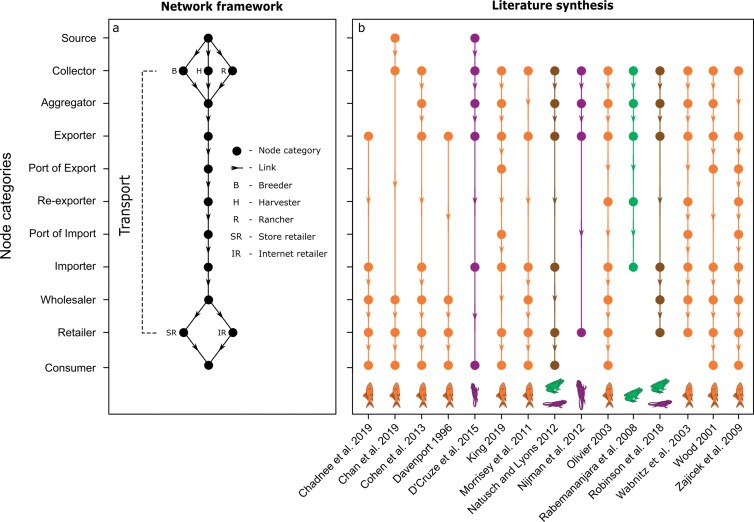
A general framework of (a) the different node categories and links in the international pet trade network, which was informed by (b) our review of publications that describe the general actors and connections involved in the legal trade of vertebrates as pets. The publications we reviewed were focused on the trade in amphibians (green), fishes (orange), reptiles (purple), or amphibians and reptiles (brown). We found no studies on birds or mammals that met our review criteria (see supplemental file S1). Our network framework outlines that pet transport (panel a, dashed line) is a directional, multistage process with pets moved from one category of nodes to the next in the chain as they are collected, transported, and eventually purchased. We also identified different subcategories of nodes from our review, such as pets collected for breeding versus ranching or store versus online retailers. However, different subcategories likely exist for all stages. Not all international trade will involve all node categories and some or many nodes can be skipped, such as pets sold directly from exporters to consumers.

Our systematic review of the wildlife trade literature provided a general structure of the pet trade commodity chain by identifying 11 broad types of actors or node categories that harbor or transport pets for international trade (see figure 1). Below, we review each of these different node categories and the links between them.

Source and collector. Animals for the pet trade are first collected from their native habitats (D'Cruze et al. 2015, Chan et al. 2019). Although many animals are bred in captivity, the first point of entry of a species into the pet trade can always be traced back to a wild population. There have also been some reported instances of captive-breeding facilities using wild-caught animals to maintain captive population viability (Warwick 2014, Tensen 2016) or illegally reporting wild-caught animals as being bred in captivity (Nijman et al. 2012, Warwick 2014).

Animals collected from the wild tend to flow into three different subcategories of collector nodes, which are commonly termed *harvesters*, *breeders*, and *ranchers* (e.g., Morrisey et al. 2011, Natusch and Lyons 2012, Harrington et al. 2020) . Harvesters do not raise or breed collected animals prior to transfer through the commodity chain. Ranchers raise wild-caught juveniles until they can be sold as adults. Breeders collect animals to start or maintain their breeding stock, with most bred offspring sold into the market.

Past research suggests that the geographic locations for collector nodes are likely determined by the native range of the species and by which parts of their geographical range are the easiest for collectors to access, such as those closest to access roads (Ceballos and Fitzgerald 2004, Gravuer et al. 2008). The strength of the links between the source and collector node categories—that is, how many animals are collected from the wild—is also influenced by customer demand and the resulting exporter demand (Robinson et al. 2018), by population density and the seasonal trends of the targeted species (Ceballos and Fitzgerald 2004, Rabemananjara et al. 2008), by the regional legality of wild harvest for the pet trade (Nijman et al. 2012), by individual-level variation in collector skill (Wood 2001, Ceballos and Fitzgerald 2004), and by animal traits (e.g., behavior; Chapple et al. 2012).

Aggregator. Once collected from the wild, animals can be transferred through intermediary aggregators prior to export (Natusch and Lyons 2012, Robinson et al. 2018). Aggregators are individuals or groups that obtain captured animals from local collectors, often from multiple locations, and consolidate these animals for eventual transfer to international exporting individuals or companies (exporters; figure 1). Likely geographic locations for aggregator nodes include areas in which target species are common—and therefore in which collection probability is higher—and areas in which transportation costs are lowest, such as regions with access roads and proximity to major cities (Rabemananjara et al. 2008). Some of the studies we reviewed also suggested that the link strength between different collectors and aggregators may be primarily influenced by the size of the exporters’ business because aggregators typically aim to fill specific orders provided by exporters (Davenport 1996, Robinson et al. 2018).

Exporter. Exporters consolidate animals from aggregators or obtain animals directly from collectors (Carpenter et al. 2005, Rosa et al. 2011). Some studies indicated that exporter nodes for the legal trade may tend to be in both the source region of collected animals and close to transport hubs, such as international airports. For example, most Indonesian exporters that specialize in trading geckos are located on Java, where the animals are primarily harvested (Nijman et al. 2012), which improves accessibility, reduces transport mortality, and is proximate to major ports for ease of international air transport. The number of animals that ultimately flow into each exporter node is determined by a combination of factors, including the size of business (Wood 2001), the quantity and mortality of animals provided by collectors or aggregators (Wood 2001, Robinson et al. 2018), the demand for pets from customers and import partners (Rabemananjara et al. 2008), and legal restrictions placed on the exporters by the exporting or recipient country (Natusch and Lyons 2012, Robinson et al. 2018).

Ports of export, reexport, and import. Once prepared for shipment by the exporter, animals are transferred to the ports of export within the source country (King 2019) . Export typically occurs via air transport but direct ground transport from the exporter to the port of import can occur between adjacent countries (e.g., Canada and the United States; Smith et al. 2017). During transport, animals can be moved to an intermediate location and reexported. At this location, multiple shipments can be consolidated and sometimes mixed with animals derived from other geographical locations, then divided into new shipments and reexported to their intended ports of import (Olivier 2003).

Importer. Following arrival at the port of import within the importing country and passage through customs inspection (if any), animals are dispersed from the port of import to consolidating locations owned by the importers (Olivier 2003, King 2019). Here, the animals are unpacked, examined, and acclimated to their new holding facilities. Importers may tend to be geographically located near international airports (Zajicek et al. 2009), but these locations can be difficult to pinpoint because registered business addresses do not necessarily reflect where the animals are being housed. The strength of the links between a given port and importer operation were not discussed in any of the studies we reviewed. However, we infer that, like exporters, these links may be primarily controlled by the size of the company and the severity of legal restrictions and enforcement, which would be imposed by the importing country and the port of import.

Sale (wholesaler, retailer, and consumer). After import, the pet trade commodity chain concludes when animals are eventually sold and housed as pets with a consumer. Sale can occur directly from importers to consumers, or animals can pass through the domestic-breeding market and through various stages of domestic transport as they are transferred from importer to wholesalers, to brick-and-mortar and internet retailers, and finally to consumers (Morrisey et al. 2011, Chan et al. 2019). Wholesalers may tend to be located near ports of import (Zajicek et al. 2009) because they purchase large quantities of pets from earlier actors in the commodity chain (often importers) for resale to retailers or consumers. However, the location of most retail stores and pet owners is likely more geographically dispersed and may be clustered in urban centers (Ceballos and Fitzgerald 2004).

## Large data gaps exist for most nodes and links in the trade network

Our second review, aimed at identifying data gaps, highlighted the sheer quantity of empirical information missing on each network component in the framework detailed above (see box 2), which limits our ability to understand and manage the pet trade (Cohen et al. 2013, Rhyne et al. 2017, Smith et al. 2017). Data availability varied widely for each network node category and their respective links. Empirical information was only broadly available for two nodes: the likely region from which animals were collected and the locations of pet retailers. Even focusing on these two parts of the network is still problematic for drawing broader conclusions about where animals came from and where they are transported. The greater data availability on pet source regions, for example, was primarily due to publicly available biodiversity databases. However, distribution databases were not created to track the pet trade and so cannot be used to infer the number of individuals collected, whether a pet was wild caught or captive bred, nor mortality associated with collection. Such knowledge gaps are becoming increasingly urgent to fill as the pet and wildlife trade affects an ever-growing proportion of global biodiversity (Di Minin et al. 2019, Scheffers et al. 2019, Marshall et al. 2020).

**Box 2. Pet trade data availability.**

We found that data availability varied widely for each network component in the international pet trade. Empirical information was generally available for the earliest node categories and links, and then became progressively less available when pets are transferred through a multitude of intermediaries. Empirical information increased for the export and import node categories and links owing to legal requirements related to the transport of legally protected species, such as the Convention on International Trade in Endangered Species of Wild Fauna and Flora (CITES). After import into a country, data availability declined dramatically again as animals were transferred through multiple intermediaries prior to sale as pets. Our conclusions of data availability for each node and link in the international pet trade network are provided in the table below (see supplemental file S1 for methods). Common data sources, proxies, or methods reported by relevant publications, and example publications, are respectively listed under the “Example sources or proxies” and “Example publications” headings. We limited the example publications to a maximum of three and so the number of references does not reflect differences in data availability (see supplemental file S1 for full references). Interviews were commonly used to obtain data across every component, and so this method is not listed as an example data source to limit repetition.

**Table:** Data availability for each node and link in the international pet trade network.

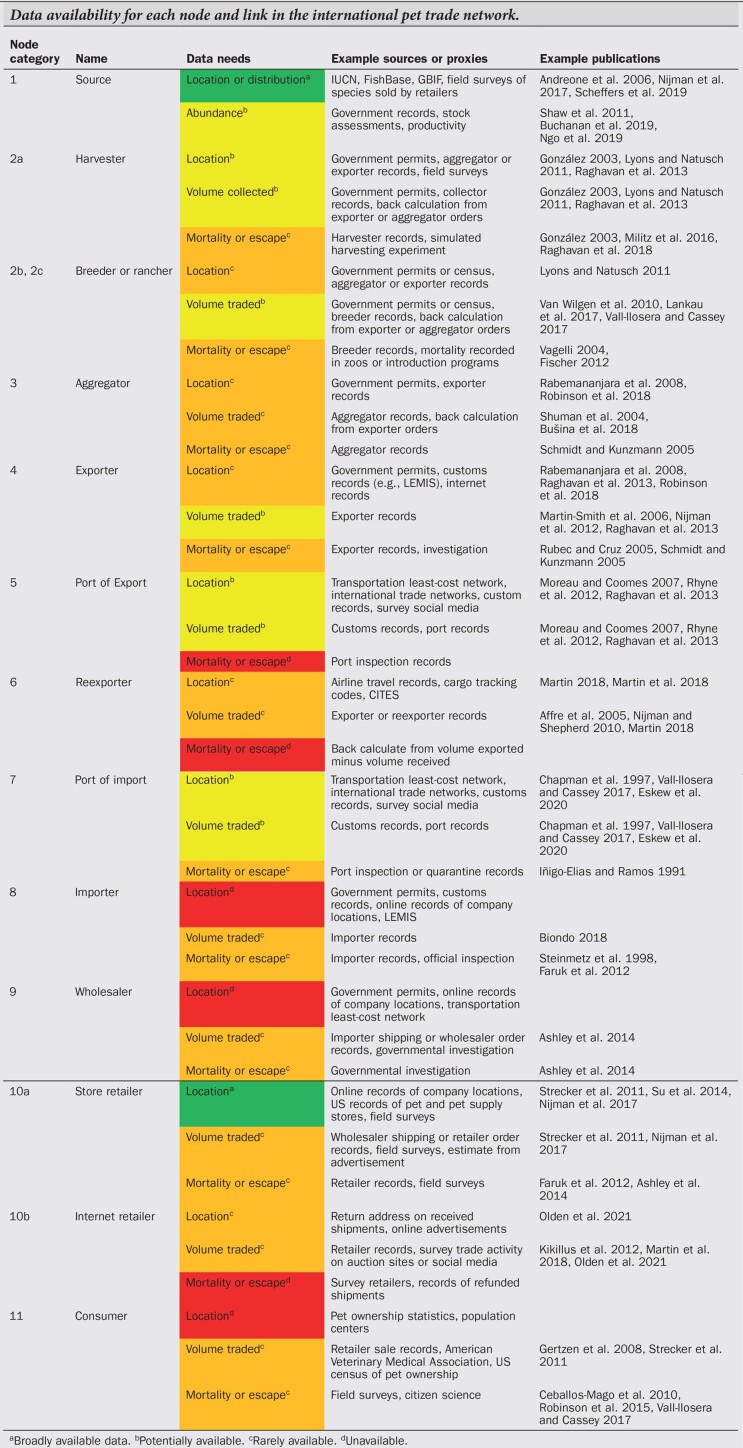

Regarding retail locations, the greater information availability for this node category was due to publicly available, online, and governmental information on brick-and-mortar stores, such as from census records or social media (e.g., Strecker et al. 2011, Stringham et al. 2020). This type of data could be used as a proxy to estimate how animals are distributed within a country if pet consumers follow similar patterns to other types of retail customers and tend to shop close to where they live (Huff 1963). However, location records generally do not provide more proprietary information on store sales. Without sales information, retail location records can only be loosely linked to the likelihood of zoonotic disease transmission or nonnative establishment. These risks would be better informed by data on the types of animals being sold (e.g., amphibians, birds), the quantity sold, and where they are sold. A greater proportion of the wildlife trade is also shifting to online markets (Lavorgna 2014), which are not captured by data on brick-and-mortar retail locations.

## US trade data illustrates the value of a network approach to pet-trade impact mitigation

Although there is a disheartening prevalence of data gaps throughout the pet trade network, relevant insights can still be gained by collecting empirical data and conducting network analyses on key components. To illustrate this point, we analyzed over 230,000 shipments of live, wild-caught and captive-bred vertebrates—exotic amphibians, birds, freshwater and marine fishes, and reptiles—that were legally imported to the United States during 1999 through 2013. This data set was obtained from the US Fish and Wildlife Service's Law Enforcement Management Information System (LEMIS) and was curated to only include shipments that could be reliably attributed to the pet trade (see supplemental file S2 for the methods). The finalized data set included 38,485 amphibian, 11,210 bird, 5667 mammal, 38,974 freshwater and marine fish, and 89,720 reptile shipment records that together detailed the trade of over 187 million individual animals over 15 years. This pet trade data is not without its limitations as it is restricted to imports into a single country and some shipments had to be excluded owing to data entry errors (which are further described in Rhyne et al. 2012 and Eskew et al. 2020). However, through an analysis of spatial and temporal patterns in US pet exports and imports (methods detailed in supplemental file S3), we show that even limited data on the trade network can help to direct future research and management approaches.

**Figure 2.**
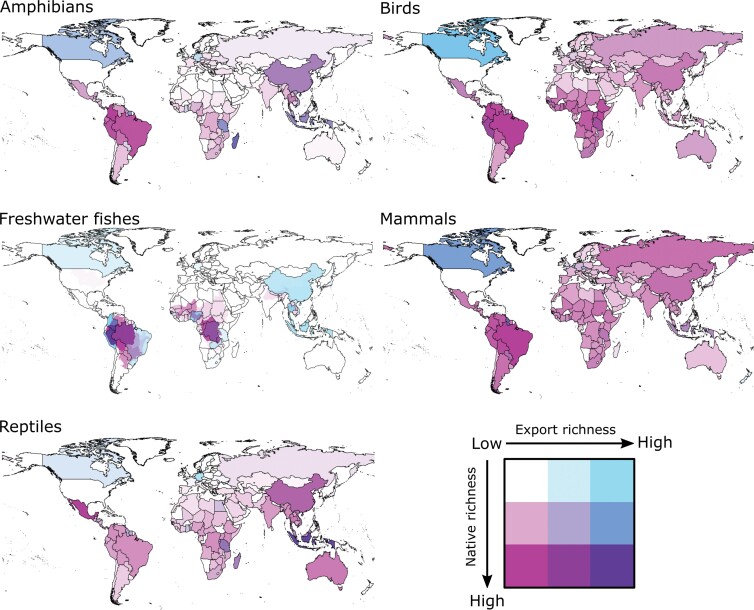
Average species richness of pet amphibians, birds, freshwater fishes, mammals, and reptiles exported from each country to the United States during 1999 through 2013 (blue; increasing values are illustrated using an increasing color gradient) overlaid on the total richness of species native to that country that are recorded in the US pet trade (pink). Richness of traded freshwater fishes is not available by country and is instead provided across global watersheds. Marine fishes are excluded from this analysis because export values are country specific and cannot be overlaid on ocean distributions. The methods for producing these maps and links to online, interactive versions are provided in supplemental file S3.

Finding hotspots of biodiversity loss and economic dependence. Our US case study illustrated how empirical data on pet exports can inform efforts to improve the economic benefits of the trade, and mitigate its biodiversity impacts, by identifying trade hotspots—that is, regions of high biodiversity loss and economic dependence. We found that just a handful of countries accounted for most of the species richness (53%–66%; table 1) and quantity (60%–97%; table 1) of animal clades exported to the United States for sale as pets. A further subset of these countries both export and harbor a high diversity of traded species (see table 2 and figure 2). For example, 95% of the amphibians exported out of Madagascar and into the United States are native to Madagascar. Such countries may also have a higher percentage of their population living in relative poverty and who depend on the pet trade as their primary income (e.g., Madagascar or Indonesia; Rabemananjara et al. 2008, Ferse et al. 2012). These local collectors are also generally paid a small fraction of the retail value of traded pets (e.g., Rabemananjara et al. 2008, Natusch and Lyons 2012, Raghavan et al. 2013). It is this combination of high native biodiversity, economic dependence on animal collection, and poor compensation that may create a trade hotspot because harvest is tightly coupled to the growing demand for new pets (Romagosa 2014, Scheffers et al. 2019, Marshall et al. 2020), and there is little monetary incentive to harvest sustainably (Wood 2001, Natusch and Lyons 2012). We cannot determine from our data whether unsustainable harvest is occurring in each country nor which traded animals are sourced from captive breeding programs versus wild capture. However, our results show how export data can help to identify potential trade hotspots. These hotspots can then be confirmed in follow-up analyses and can be targeted for livelihood diversification and equitable benefit sharing (e.g., Ferse et al. 2012, Hinsley et al. 2018), and ultimately culturally appropriate interventions to reduce the biodiversity impacts of the trade (e.g., Agu and Gore 2020).

**Table 1.** Top five exporting countries and importing ports in the US trade of pet amphibians, birds, fishes, mammals, and reptiles during 1999 through 2013.

Top five export or import	Amphibians	Birds	Fishes	Mammals	Reptiles
Average export richness	Madagascar (20.5)	Canada (56.1)	Indonesia (17.0)	Guyana (9.1)	Indonesia (89.3)
	Tanzania (15.1)	Belgium (23.3)	Peru (11.1)	Canada (8.3)	Germany (60.9)
	Suriname (12.8)	Peru (18.7)	Colombia (10.9)	Czech Republic (6.8)	Tanzania (53.2)
	Indonesia (12.7)	Suriname (17.3)	Thailand (10.1)	Netherlands (6.4)	Madagascar (39.2)
	Canada (12.2)	Tanzania (14.8)	Philippines (9.6)	Indonesia (3.5)	Guyana (31.5)
Percent of total export richness	62%	57%	53%	58%	66%
	(203 of 326)	(375 of 652)	(155 of 290)	(86 of 147)	(745 of 1119)
Average export quantity	Hong Kong (709K)	Taiwan (29K)	Trinidad and Tobago (3.1M)	Netherlands (48K)	Vietnam (302K)
	Singapore (414K)	Tanzania (25K)	Thailand (1.8M)	Czech Republic (42K)	El Salvador (191K)
	China (383K)	Belgium (22K)	China (1.4M)	Canada (8K)	Colombia (94K)
	Indonesia (188K)	Senegal (22K)	Hong Kong (1.3M)	Peru (1K)	Togo (86K)
	South Korea (97K)	Australia (15K)	Malaysia (354K)	Indonesia (1K)	Indonesia (78K)
Percent of total import quantity	87%	62%	90%	97%	60%
	(2.7M of 3.1M)	(1.7M of 2.7M)	(12.0M of 13.3M)	(1.5M of 1.54M)	(11.2M of 18.7M)
Average import richness	Miami (84.1)	Los Angeles (103.2)	Houston (33.6)	Miami (16.5)	Miami (363.1)
	Los Angeles (67.0)	Miami (64.9)	New York (29.8)	Dallas–Fort Worth (14.1)	Los Angeles (227.3)
	Dallas–Fort Worth (14.1)	New York (32.4)	Los Angeles (13.8)	Chicago (7.1)	Dallas–Fort Worth (92.1)
	New York (13.5)	Buffalo (27.1)	Atlanta (13.0)	Los Angeles (5.7)	New York (38.0)
	Atlanta (5.7)	Detroit (13.5)	San Francisco (12.7)	Houston (3.8)	Atlanta (25.9)
Percent of total import richness	89%	91%	81%	86%	95%
	(289 of 326)	(594 of 652)	(235 of 290)	(127 of 147)	(1066 of 1119)
Average import quantity	Los Angeles (1.4M)	Los Angeles (150K)	New York (3.9M)	Dallas–Fort Worth (52K)	Miami (673K)
	New York (168K)	New York (18K)	San Francisco (2.1M)	Chicago (16K)	Los Angeles (487K)
	Tampa (141K)	Miami (6K)	Los Angeles (1.7M)	Atlanta (9K)	Dallas–Fort Worth (45K)
	Miami (116K)	Pembina (5K)	Atlanta (338K)	Los Angeles (9K)	New York (11K)
	Chicago (106K)	Buffalo (1K)	Tampa (181K)	New York (6K)	Baltimore (7K)
Percent of total import quantity	96%	99%	93%	91%	98%
	(3.0M of 3.1M)	(2.7M of 2.72M)	(12.4M of 13.3M)	(1.4M of 1.5M)	(18.3M of 18.7M)

*Note:* Trade richness is the average number of species exported or imported across years (species per year), and quantity is the average number of animals (individuals per year). The percentage of total trade richness (species) or quantity (individuals) represented by the top five countries or ports across all years combined is also provided for each animal clade. The quantity values are rounded to the nearest 1000, 10,000, or 100,000, depending on trade magnitude.

**Table 2.** Agreement between the number of species exported from a given country to the United States and how many species in the pet trade naturally occur within that country for each animal clade.

Match between native and export richness	Amphibians	Birds	Mammals	Reptiles
High export and high diversity (total species exported of total native species in trade)	Madagascar	Peru	Indonesia	Indonesia
	(42 of 38)	(74 of 132)	(17 of 19)	(200 of 147)
	Indonesia	Tanzania	Guyana	Tanzania
	(41 of 29)	(73 of 128)	(16 of 32)	(144 of 113)
	Suriname	Guinea	Ghana	China
	(41 of 28)	(63 of 103)	(11 of 19)	(88 of 125)
Low export and high diversity	Colombia	Columbia	Bolivia^a^	Mexico
	(5 of 52)	(10 of 143)	(0 of 30)	(32 of 162)
	Brazil^a^	Brazil	Brazil	Myanmar
	(0 of 45)	(13 of 146)	(3 of 32)	(1 of 96)
	Bolivia^a^	Kenya	Venezuela^a^	Brazil
	(0 of 38)	(3 of 134)	(0 of 27)	(3 of 97)
High export and low diversity	Germany	Canada	Canada	Germany
	(43 of 8)	(186 of 31)	(39 of 13)	(380 of 8)
	Canada	Belgium	Czech Republic	Canada
	(42 of 14)	(78 of 40)	(34 of 11)	(138 of 23)
	Netherlands	Netherlands	Netherlands	Netherlands
	(29 of 5)	(73 of 38)	(25 of 10)	(70 of 4)

*Note:* Countries with a high diversity of exports and a high diversity of native species in the pet trade (purple countries in figure 1) are potential trade hotspots. Countries with low export richness but a high richness of native species in the trade (pink countries in figure 1) are potential source regions of pets reexported, bred, or smuggled in other countries. Countries with much higher export richness than their native richness (blue countries in figure 1) are potentially prolific breeders or reexporters. The top three such countries are listed in each table subsection. ^a^Countries that are never recorded as the country of origin for a given animal clade in the LEMIS database despite having a high diversity of native species present in the pet trade.

Reducing disease transmission. A network approach can also inform mitigation efforts aimed at controlling wildlife-associated disease spillover. Knowing the location of the source nodes (figure 1) aids in the identification of which diseases are potentially being moved with their animal hosts (Springborn et al. 2015), as can information on wild capture versus captive breeding (Smith et al. 2017). Furthermore, high volume reexport and import nodes can consolidate closely related animals from multiple source regions under stressful transport conditions, which may aid the transmission of pathogens between individuals and species (Warwick 2014). Knowing the principal reexporters and ports of import can therefore greatly aid biosecurity enforcement aimed at preventing such transmissions through inspections and quarantines.

**Figure 3.**
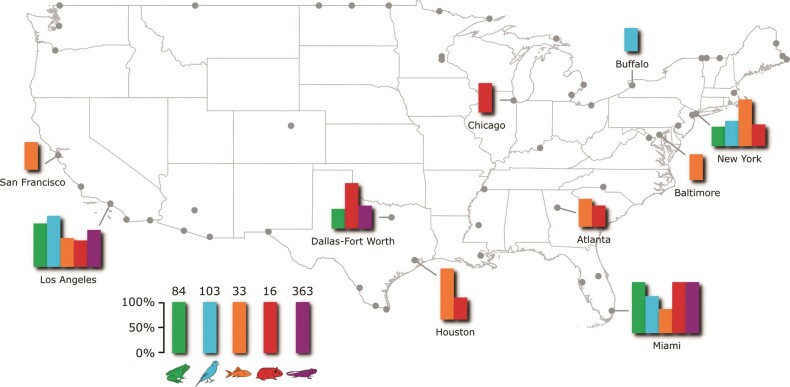
Average species richness of amphibians (green), birds (blue), fishes (orange), mammals (red), and reptiles (purple) imported to each US port of entry (air and land ports) for sale as pets during 1999 through 2013. There are strong spatial patterns in where different animal clades tend to be imported into the United States such that only a handful of ports—primarily the Dallas–Fort Worth, Los Angeles, Miami, and New York airports—import the majority of traded species (these same four ports also import the greatest quantities; see supplemental file S3). Bars are scaled relative to the maximum import richness for each animal clade, which is listed at the top of each bar in the legend. Ports whose imports constitute less than 5% of maximum import richness, and are therefore not principal ports of entry for imported pets, are plotted as grey points.

**Figure 4.**
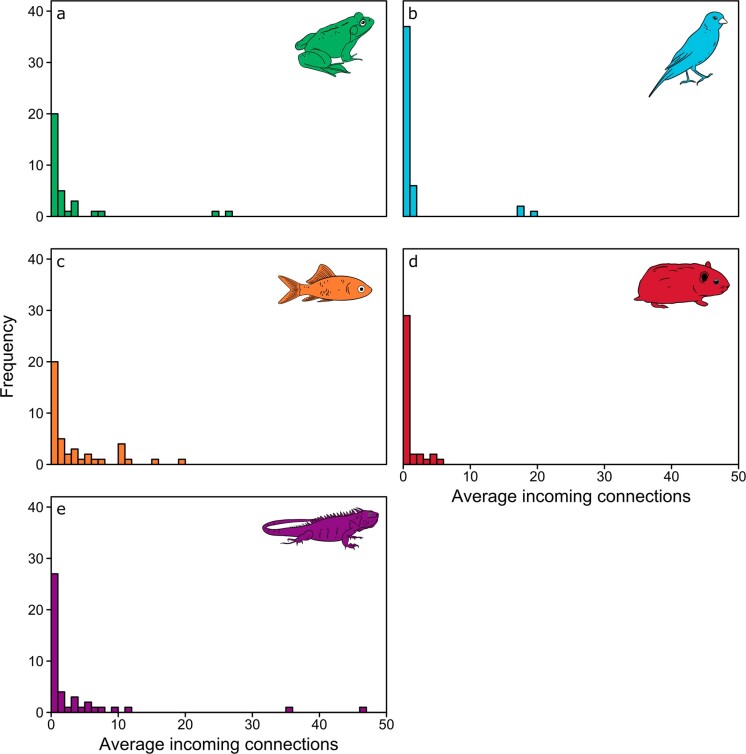
Frequency distributions of incoming trade connections for pet (a) amphibians, (b) birds, (c) fishes, (d) mammals, and (e) reptiles imported to each US port of entry. The incoming connections for each port are calculated as the number of different countries a port receives shipments from for each animal clade averaged from 1999 through 2013. These right-skewed distributions indicate that most ports tend to import pets from just a single country, whereas a small number of ports (usually the Los Angeles, Miami, or New York airports) import and therefore consolidate pets from many different countries. Histograms of outgoing connections are similarly right skewed and are provided in supplementary materials S3.

**Figure 5.**
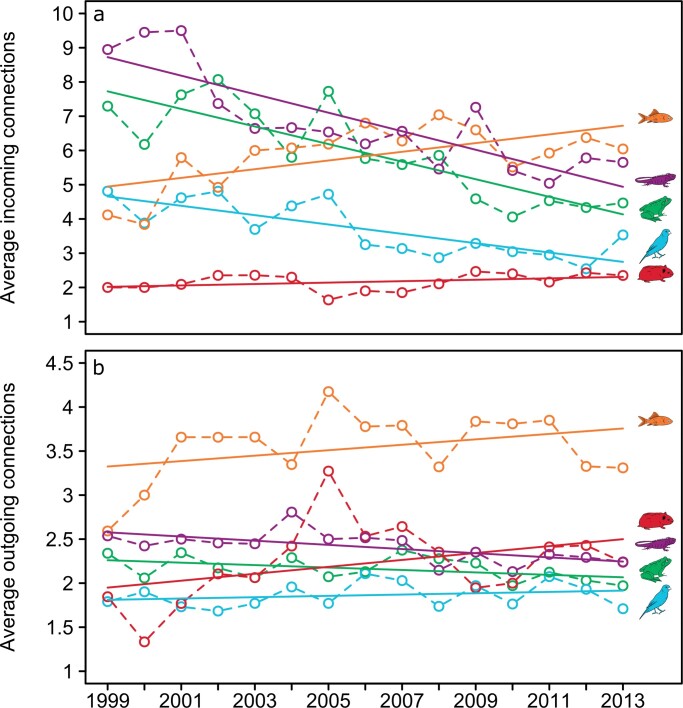
Temporal changes in the average number of (a) incoming and (b) outgoing trade connections during 1999 through 2013 for each animal clade. Both incoming and outgoing connections have declined over time for amphibians (green) and reptiles (purple), indicating a spatial consolidation of trade, whereas these metrics have increased in fishes (orange) and mammals (red). For birds (blue), incoming connections have decreased over time, whereas outgoing connections have increased. Best-fit lines are plotted for each animal clade on the basis of their linear relationship with time predicted from ANCOVA models (see supplementary materials S3).

For example, our case study showed that four US ­airports—Dallas, Los Angeles, Miami, and New York—­consistently received the highest richness (table 1 and figure 3) and quantity (table 1) of pet imports. Intensifying enforcement of biosecurity policy at these large animal import hubs could have a strong influence on mitigating disease introductions (e.g., van Roon et al. 2019). In addition, our network analyses of incoming and outgoing trade connections indicated that just a few US ports were responsible for importing pets from a very high number of source countries, and a few countries spatially dispersed their animal exports to a very high number of US ports. These trade patterns were evidenced by strongly right-skewed distributions of trade connections (figure 4). Our network results also showed that the US trade in amphibian and reptile pets has spatially consolidated over time, whereas the trade in fishes and mammals has become more spatially dispersed (on the basis of the data available during 1999 through 2013; figure 5). Exporters and animal clades that are creating broader spatial distribution, such as Canada, which exported birds to an average of 13 different US ports across years, could be important targets for increased enforcement because greater distribution can facilitate more widespread disease introduction (Shirley and Rushton 2005). Conversely, ports and animal clades that are spatially consolidating can help to simplify biosecurity to a small number of target ports that warrant higher staffing and monitoring by inspectors (Floerl et al. 2009, Silk et al. 2017).

Preventing nonnative introductions and revealing regions of high invasion risk. Efforts to prevent nonnative species introductions via the pet trade can also benefit from our network approach. Identifying the principal countries of export (e.g., Canada, Indonesia, or Madagascar in the US trade; table 1) and ports of import can provide key targets for mitigation efforts aiming to prevent new species introductions (Reaser et al. 2008). In addition, information on nonnative pet origins (i.e., the source nodes), and distribution within recipient countries from importer to consumer (figure 1), can be used to inform predictions of environmental matching between native and potential nonnative distributions (Howeth et al. 2016). For instance, some commonly traded amphibian, bird, and reptile species that we found in the US trade tend to be native to warmer climates, such as the western dwarf clawed frog (*Hymenochirus curtipes*), the savannah monitor (*Varanus exanthematicus*), and the estrildid finches (family Estrildidae). These same animals also tended to be imported to the warmer regions of the United States (e.g., Miami and Los Angeles). We do not know whether they were subsequently sold as pets in these warmer regions, but a high environmental match between the source and import regions suggests a higher risk of successful establishment.

Finally, although data are scarce, a better understanding of the later nodes in the commodity chain (i.e., retailers and consumers) is critical for predicting pet escape or release, which are major pathways driving the establishment of new nonnative animals worldwide (Hulme et al. 2008, Stringham and Lockwood 2018). Although our US case study did not extend to these later nodes, the differences we found in animal import patterns provides an example of potential differences in spatial distribution that could be informative of establishment risk. We found that the majority of amphibians, birds, and reptiles were imported via Miami and Los Angeles, the majority of freshwater and marine fishes were imported via New York and Dallas, and the majority of mammals were imported to Miami and Dallas (figure 3). These spatial import patterns may result in some regions receiving more introductions of particular animal clades, such as more reptiles being introduced to the southeastern and southwestern United States, which can translate to a higher invasion risk (Lockwood et al. 2005).

Identifying potential breeders, reexporters, or sources of illegally traded pets. Even network approaches using incomplete trade data, such as the LEMIS shipment records that are not designed to track the pet trade, can inform management efforts by helping to locate key network components, such as prolific reexporters, breeders, or possibly routes of illegal wildlife trade. In our US case study, for example, we found a variety of mismatches in which countries were exporting more species than they possessed as native residents (table 2). Such mismatches could indicate data deficiencies where the reported country of origin in export and import records may not reflect the actual source region of traded animals, providing insight into potentially important nodes and trade links. For instance, Canada, Germany, and the Netherlands were often listed as the region of origin of a diverse array of animals not native to any of these countries, which suggests that these countries are likely home to a variety of captive breeding programs or are prolific reexporters. This information can subsequently inform management efforts depending on the source of the traded animals and the conditions of captivity. If these countries are breeding different species for sale as pets then they may be low priority management targets because well-managed breeding facilities can result in lower biodiversity and disease impacts (Smith et al. 2009). However, poorly managed programs may be high priority targets owing to stressful and crowded conditions that can increase disease susceptibility and transmission (Warwick 2014). Alternatively, if the exporting countries are prolific reexporters then they are potentially facilitating the harvest of animals and consolidation of wildlife diseases from a variety of source regions. These types of countries may therefore be important targets for enforcement of inspection and quarantine regulations and for determining the origins of their shipments.

Other mismatches occurred in our US case study in which some countries harbored a high diversity of traded species but were rarely listed as the countries of origin in any shipment records, such as Brazil, Columbia, and Bolivia (table 2). It is possible that, although a variety of traded species naturally occur in these regions, traded animals could be mostly sourced from neighboring countries in which they also naturally occur or are being sourced from captive-breeding programs. Alternatively, countries that harbor a high diversity of traded species but are not themselves listed as the origin of these shipments could be the unreported source regions for many traded pets being shipped or smuggled to intermediate countries prior to import into the United States (Nóbrega Alves et al. 2013, Patoka et al. 2015). This form of reexporting may appear legal if significant time is spent in the layover country or if breeding facilities are established, effectively laundering the initial wild source region of these animals. Such hidden source countries may therefore be unrecognized trade hotspots or overlooked actors in the illegal trade of wild-caught pets, which can be revealed through network approaches and by filling in data gaps in the pet trade network.

## Conclusions

The vertebrate pet trade is a poorly understood but increasingly important driver of the translocation of millions of animals around the world, presenting serious conservation and public health concerns. However, this same trade offers myriad positive economic and social benefits ranging from supporting local economies to providing emotional companionship. Here, we show that developing conceptual and empirical network representations of the pet trade allows targeted interventions that mitigate its negative impacts and could improve its positive economic benefits. Our literature synthesis revealed that the full pet trade network is a primarily directional, multistage process, and empirical data are sorely lacking for most stages. The complexity and data gaps of the trade network stem from animals transferring through many different entities and locations on their journey from their source habitats to a consumer. Encouragingly, our case study demonstrates that even networks sparsely informed by empirical data can provide important insights into the potential spatial and temporal patterns of the pet trade, such as inferring the initial geographic origins and eventual distribution of purchased animals. These insights provide a better understanding of the pet trade, which is necessary to improve the management of its impacts, to identify the people that depend on it financially, and to ensure it is conducted sustainably so that it can continue to provide its beneficial services.

## Acknowledgments

We would like to thank Marie-Josée Létourneau for producing the animal artwork used in some of our figures and for agreeing to its publication. This work was supported by the National Socio-Environmental Synthesis Center under funding received from the US National Science Foundation (grant no. DBI*‐*1052875) and with project funding from the Centre for Invasive Species Solutions (grant no. PO1*‐*I*‐*002: “Understanding and intervening in illegal trade in non*‐*native species”).

## Supplementary Material

biab056_Supplemental_Files

## Author Biographical

James Sinclair (james.sinclair270@gmail.com) is a postdoctoral researcher with the Aquatic Ecology Lab at The Ohio State University, in Columbus, Ohio, in the United States. Oliver Stringham is a postdoctoral researcher with the Invasion Science and Wildlife Ecology Lab at The University of Adelaide, in Adelaide, South Australia, in Australia. Bradley Udell is a former PhD student at the University of Florida, in Gainesville, and is currently an ecologist with the US Geological Survey's North American Bat Monitoring Program at the Fort Collins Science Center, in Fort Collins, Colorado, in the United States. Nicholas Mandrak is a professor at the University of Toronto Scarborough, in Toronto, Ontario, in Canada. Brian Leung is an assistant professor at McGill University, the UNESCO Chair for Dialogues on Sustainability, and the Director of the McGill-STRI Neotropical Environment Option, in Montreal, Quebec, in Canada. Christina Romagosa is an associate professor at the University of Florida, in Gainesville, Florida, in the United States. Julie Lockwood is a professor and chair of the Department of Ecology, Evolution, and Natural Resources at Rutgers University, in New Brunswick, New Jersey, in the United States.
