# Supplementary material for: The International Vertebrate Pet Trade Network and Insights from US Imports of Exotic Pets
Source: Bioscience. 2021 Jun 9;71(9):977–90. doi: 10.1093/biosci/biab056 (PMC8407969; doi:10.1093/biosci/biab056)
Supplement: biab056_Supplemental_Files [file biab056_supplemental_files.zip › Supplementary_Materials_S1.docx]

**Supplementary Materials S1** – Literature reviews

Our first literature review was conducted in January 2020, which used search terms designed to capture articles that detail the structure, stages, or trade flows involved in the international vertebrate pet trade (see table S1.1 for search terms). Searches were conducted using Web of Science (WoS), with results limited to the ‘Ecology’, ‘Zoology’, and ‘Infectious diseases’ categories. Additional searches were also conducted using Google Scholar and citations in relevant papers to identify additional articles that were not included in our WoS search. Our searches returned a total of 496 articles, of which 465 came from WoS and an additional 31 from Google Scholar or paper citations (see figure S1.1).

We curated our search results to the international pet trade commodity chain by only including results that encompassed: (i) the legal pet trade (e.g., not trade of food animals, animal parts, derived products, or trafficked species); (ii) trade in live vertebrates; (iii) international transport; and (iv) trade between actors beyond just exporters and importers, which is already well characterized in the literature (e.g., Bush et al. 2014, Reino et al. 2017). From our total pool of search results, 363 were excluded due to a lack of relevance, such as papers on diseases in domesticated pets, or because they provided no assessment of trade activity, such as studies of pet ownership patterns (e.g., Alves et al. 2019). A further 83 studies were excluded because they were not specific to the legal pet trade, such as those involving trade in general wildlife (e.g., Nijman and Shepherd 2010), animal products (e.g., Bachmann et al. 2019), or trafficking (e.g., Sánchez-Mercado et al. 2020). An additional 3 studies were excluded because they detailed trade in invertebrates (e.g., Patoka et al. 2015), and 32 studies were excluded because they did not detail international transport (e.g., Maceda-Veiga et al. 2013) or were limited to country-level export/import connections (e.g., Martin 2018). After these exclusions, 15 publications remained which we used to inform our composite pet-trade network. From synthesizing these articles, we identified 11 different general types of actors or ‘node categories’ that harbor and/or transport pets for international trade (see figure 1 and associated references below).

We conducted a second systematic review of publications and databases to assess the availability of information for each type of node and associated links that our initial review identified within the pet trade network (results are detailed in box 1). This second review was tailored toward finding empirical information on the geographic locations of each node, the link strength between nodes (i.e., volume traded), and mortality or escape rates within and between nodes (see table S1.2 for search terms). We considered these elements as being the principal information required to construct a spatially explicit model of the pet trade network and its potential economic, ecological, ethical, and human health impacts. Each node type, link, and mortality or escape assessment was qualitatively assigned to one of four information availability categorizations: (i) ‘broadly available’ if multiple publicly available datasets were found for entire animal clades (e.g., fishes in FishBase); (ii) ‘potentially available’ if several publications were found that provided species-specific datasets; (iii) ‘rarely available’ if only one or two publications could be found with species-specific data; and, (iv) ‘unavailable’ if no publications were found for a given network subcomponent.

**References**

Alves RRN, de Araújo BMC, da Silva Policarpo I, Pereira HM, Borges AKM, da Silva Vieira WL, Vasconcellos A. 2019. Keeping reptiles as pets in Brazil: Ethnozoological and conservation aspects. Journal for Nature Conservation 49: 9–21.

Bachmann ME, Junker J, Mundry R, Nielsen MR, Haase D, Cohen H, Kouassi JAK, Kühl HS. 2019. Disentangling economic, cultural, and nutritional motives to identify entry points for regulating a wildlife commodity chain. Biological Conservation 238: 108177.

Bush ER, Baker SE, Macdonald DW. 2014. Global trade in exotic pets 2006–2012. Conservation Biology 28: 663–676.

Maceda-Veiga A, Escribano-Alacid J, de Sostoa A, García-Berthou E. 2013. The aquarium trade as a potential source of fish introductions in southwestern Europe. Biological Invasions 15: 2707–2716.

Martin RO. 2018. The wild bird trade and African parrots: Past, present and future challenges. Ostrich 89: 139–143.

Nijman V, Shepherd CR. 2010. The role of Asia in the global trade in CITES II-listed poison arrow frogs: Hopping from Kazakhstan to Lebanon to Thailand and beyond. Biodiversity and Conservation 19: 1963–1970.

Patoka J, Kalous L, Kopecký O. 2015. Imports of ornamental crayfish: The first decade from the Czech Republic’s perspective. Knowledge and Management of Aquatic Ecosystems 416.

Reino L, Figueira R, Beja P, Araújo MB, Capinha C, Strubbe D. 2017. Networks of global bird invasion altered by regional trade ban. Science Advances 3: e1700783.

Sánchez-Mercado A, Cardozo-Urdaneta A, Moran L, Ovalle L, Arvelo MÁ, Morales-Campos J, Coyle B, Braun MJ, Rodríguez-Clark KM. 2020. Social network analysis reveals specialized trade in an endangered songbird. Animal Conservation 23: 132–144.

***Table S1.1. Search terms used to identify papers that detail the structure, stages or trade flows involved in the international vertebrate pet trade. The same keywords were used for both Web of Science and Google Scholar searches.***

| **Trade keywords** | | **Commodity chain keywords** |
| --- | --- | --- |
| pet | AND | (“supply chain” OR “commodity chain” OR “value chain” OR “trade route” OR “trade chain” OR “market* channel” OR chain OR intermedia* OR middlem*n OR traceability OR flowchart OR flows OR node* OR pathway OR network OR structure OR topology OR tranship* OR transship* OR “re-exporter” OR “re-export”) |
| “pet trade” | AND |  |
| “aquarium trade” | AND |  |

***Table S1.2. Search terms used to identify data gaps for each node and link of the international vertebrate pet trade. The same keywords were used for both Web of Science and Google Scholar searches.***

| **Node** |  | **Trade keywords** | + | **Stage name keywords** | **+** | **Data need keywords** |  |
| --- | --- | --- | --- | --- | --- | --- | --- |
| 1 - Source |  | (pet OR “pet trade” OR “aquarium trade”) |  | (source OR native) |  | (locat* OR distribution OR abundance OR volume) |  |
| 2a - Breeder |  | (pet OR “pet trade” OR “aquarium trade”) |  | (breed*) |  | (locat* OR volume OR mortality OR escape*) |  |
| 2b - Harvester |  | (pet OR “pet trade” OR “aquarium trade”) |  | (collect* OR harvest*) |  | (locat* OR volume OR mortality OR escape*) |  |
| 2c - Rancher |  | (pet OR “pet trade” OR “aquarium trade”) |  | (ranch*) |  | (locat* OR volume OR mortality OR escape*) |  |
| 3 - Aggregator |  | (pet OR “pet trade” OR “aquarium trade”) |  | (aggregat* OR middlem*n OR intermediary) |  | (locat* OR volume OR mortality OR escape*) |  |
| 4 - Exporter |  | (pet OR “pet trade” OR “aquarium trade”) |  | export* |  | (locat* OR volume OR mortality OR escape*) |  |
| 5 – Port of export |  | (pet OR “pet trade” OR “aquarium trade”) |  | (export* AND port) |  | (locat* OR volume OR mortality OR escape*) |  |
| 6 – Re-exporter |  | (pet OR “pet trade” OR “aquarium trade”) |  | (transship* OR tranship* OR “trans-shipper” OR reexport* OR “re-export” OR “re-exporter” OR “re-exported”) |  | (locat* OR volume OR mortality OR escape*) |  |
| 7 – Port of import |  | (pet OR “pet trade” OR “aquarium trade”) |  | (import* AND port) |  | (locat* OR volume OR mortality OR escape*) |  |
| 8 - Importer |  | (pet OR “pet trade” OR “aquarium trade”) |  | importer |  | (locat* OR volume OR mortality OR escape*) |  |
| 9 - Wholesaler |  | (pet OR “pet trade” OR “aquarium trade”) |  | wholesale* |  | (locat* OR volume OR mortality OR escape*) |  |
| 10 – Retailer |  | (pet OR “pet trade” OR “aquarium trade”) |  | (retail* OR store OR sale OR sold) |  | (locat* OR volume OR mortality OR escape*) |  |
| 11 - Consumer |  | (pet OR “pet trade” OR “aquarium trade”) |  | (purchase* OR buy OR bought OR sale OR sold OR customer OR consumer OR owner) |  | (locat* OR volume OR mortality OR escape*) |  |


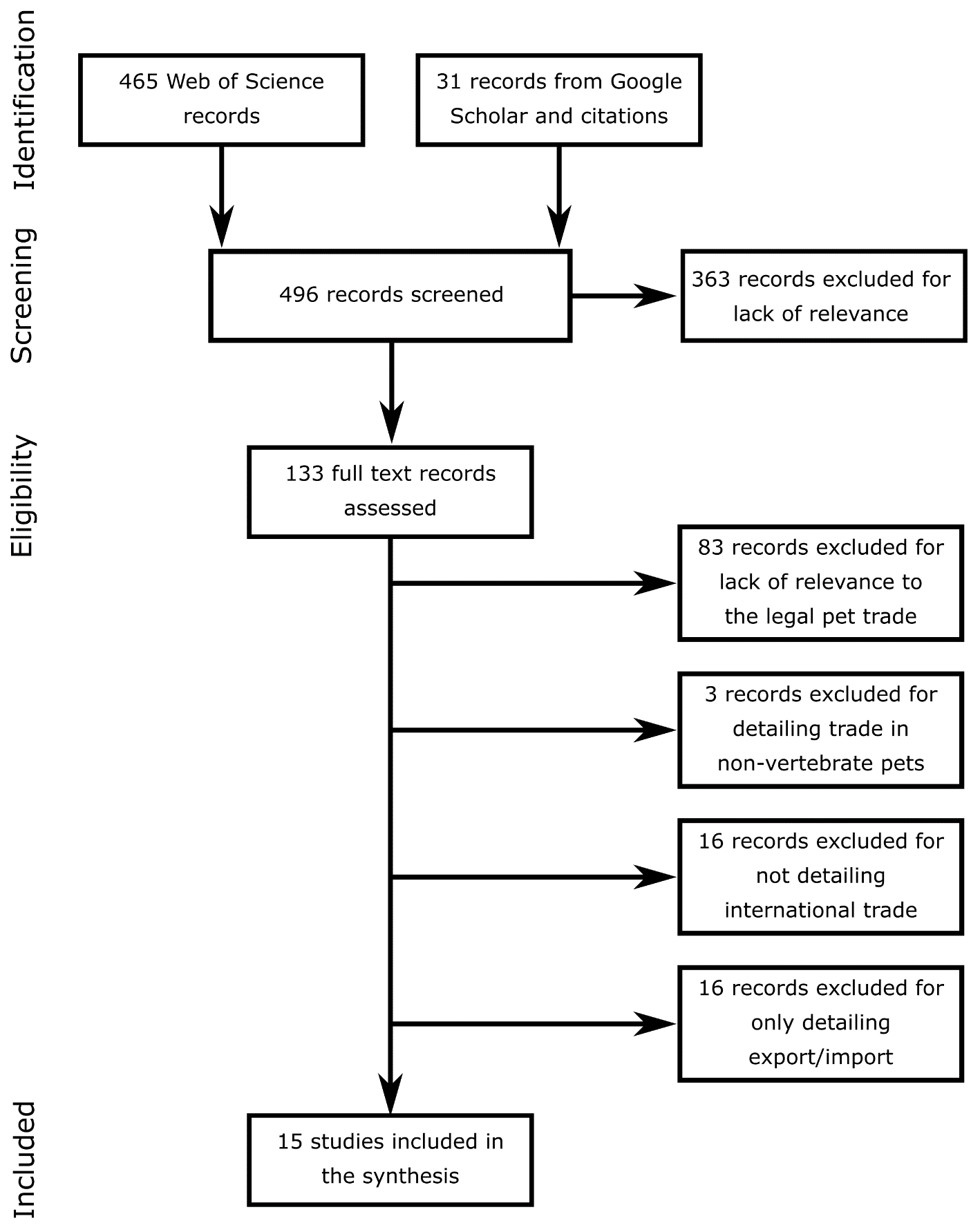


***Figure S1.1. Flow chart of the record identification process for the literature referenced in figure 2. The search terms that were used are detailed in table S1.1.***

**References for figure 1**

Chadnee FK, Uddin MS, Dey T, Hossain MDS, Akter R. 2019. Species availability and business trend of ornamental fish in Sylhet division. International Journal of Fisheries and Aquatic Studies 7:182–186.

Chan FT, et al. 2019. Leaving the fish bowl: the ornamental trade as a global vector for freshwater fish invasions. Aquatic Ecosystem Health & Management 22: 417–439.

Cohen FPA, Valenti WC, Calado R. 2013. Traceability issues in the trade of marine ornamental species. Reviews in Fisheries Science 21: 98–111.

Davenport KE. 1996. Characteristics of the current international trade in ornamental ﬁsh, with special reference to the European Union. Revue Scientiﬁque et Technique de l’Ofﬁce International des Epizoonties 15: 435-443.

D’Cruze N, Singh B, Morrison T, Schmidt-Burbach J, Macdonald DW, Mookerjee A. 2015. A star attraction: the illegal trade in Indian Star Tortoises. Nature Conservation 13: 1–19.

King TA. 2019. Wild caught ornamental fish: A perspective from the UK ornamental aquatic industry on the sustainability of aquatic organisms and livelihoods. Journal of Fish Biology 94: 925–936.

Morrisey D, Inglis G, Neil K, Bradley A, Fitridge I. 2011. Characterization of the marine aquarium trade and management of associated marine pests in Australia, a country with stringent import biosecurity regulation. Environmental Conservation 38: 89–100.

Natusch DJD, Lyons JA. 2012. Exploited for pets: the harvest and trade of amphibians and reptiles from Indonesian New Guinea. Biodiversity and Conservation 21: 2899–2911.

Nijman V, Shepherd CR, Mumpuni, Sanders KL. 2012. Over-exploitation and illegal trade of reptiles in Indonesia. Herpetological Journal 22: 83–89.

Olivier K. 2003. World trade in ornamental species. Pages 49-63 in Cato JC, Brown CL, eds. Ornamental Species: Collection, Culture and Conservation. Iowa State Press.

Rabemananjara FCE, Rasoamampionona Raminosoa N, Ravoahangimalala Ramilijaona O, Andreone F, Bora P, Carpenter AI, Glaw F, Razafindrabe T, Vallan D, Vieites DR. 2008. Malagasy poison frogs in the pet trade: A survey of levels of exploitation of species in the genus Mantella. Amphibian and Reptile Conservation 5: 3–16.

Robinson JE, Fraser IM, John FAV St., Randrianantoandro JC, Andriantsimanarilafy RR, Razafimanahaka JH, Griffiths RA, Roberts DL. 2018. Wildlife supply chains in Madagascar from local collection to global export. Biological Conservation 226: 144–152.

Wabnitz C, Taylor M, Green E, Razak T. 2003. From ocean to aquarium: the global trade in marine ornamental species. UNEP/Earthprint.

Wood EM. 2001. Collection of coral reef fish for aquaria: global trade, conservation issues and management strategies. Marine Conservation Society, Ross-on-Wye, UK.

Zajicek P, Hardin S, Watson C. 2009. A Florida marine ornamental pathway risk analysis. Reviews in Fisheries Science 17: 156–169.

**References for box 2 in the main text**

Affre A, Ineich I, Ringuet S. 2005. West-Africa, Madagascar, Central-and South-America: Main origins of the CITES-listed lizard pet market in France. Herpetological Review 36: 133–137.

Andreone F, Mercurio V, Mattioli F. 2006. Between environmental degradation and international pet trade: Conservation strategies for the threatened amphibians of Madagascar. Natura 95: 81–96.

Ashley S, Brown S, Ledford J, Martin J, Nash A-E, Terry A, Tristan T, Warwick C. 2014. Morbidity and mortality of invertebrates, amphibians, reptiles, and mammals at a major exotic companion animal wholesaler. Journal of Applied Animal Welfare Science 17: 308–321.

Biondo M V. 2018. Importation of marine ornamental fishes to Switzerland. Global Ecology and Conservation 15: e00418.

Buchanan SW, Buffum B, Puggioni G, Karraker NE. 2019. Occupancy of freshwater turtles across a gradient of altered landscapes. The Journal of Wildlife Management 83: 435–445.

Bušina T, Pasaribu N, Kouba M. 2018. Ongoing illicit trade of Sumatran Laughingthrush *Garrulax bicolor*: One-year market monitoring in Medan, North Sumatra. Kukila 21: 27–34.

Ceballos-Mago N, Chivers DJ. 2010. Local knowledge and perceptions of pet primates and wild Margarita capuchins on Isla de Margarita and Isla de Coche in Venezuela. Endangered Species Research 13: 63–72.

Chapman FA, Fitz-Coy SA, Thunberg EM, Adams CM. 1997. United States of America trade in ornamental fish. Journal of the World Aquaculture Society 28: 1–10.

Faruk MAR, Hasan MM, Anka IZ, Parvin MK. 2012. Trade and health issue of ornamental fish in Bangladesh. Bangladesh Journal of Progressive Science and Technology 10:163-168.

Fischer D. 2012. Notes on the husbandry and breeding of the black tree monitor *Varanus* *(Euprepiosaurus) beccarii* (Doria, 1874). Biawak 6: 79– 87.

Gertzen E, Familiar O, Leung B. 2008. Quantifying invasion pathways: Fish introductions from the aquarium trade. Canadian Journal of Fisheries and Aquatic Sciences 65: 1265–1273.

González JA. 2003. Harvesting, local trade, and conservation of parrots in the Northeastern Peruvian Amazon. Biological Conservation 114: 437–446.

Iñigo-Elias EE, Ramos MA. 1991. The psittacine trade in Mexico. Pages 380– 392 in Robinsonand JG, Redford KH, eds. Neotropical Wildlife Use and Conservation. Chicago University Press.

Kikillus KH, Hare KM, Hartley S. 2012. Online trading tools as a method of estimating propagule pressure via the pet-release pathway. Biological Invasions 14: 2657–2664.

Lankau EW, Sinclair JR, Schroeder BA, Galland GG, Marano N. 2017. Public health implications of changing rodent importation patterns – United States, 1999–2013. Transboundary and Emerging Diseases 64: 528–537.

Lyons JA, Natusch DJD. 2011. Wildlife laundering through breeding farms: Illegal harvest, population declines and a means of regulating the trade of green pythons (*Morelia viridis*) from Indonesia. Biological Conservation 144: 3073–3081.

Martin RO. 2018. The wild bird trade and African parrots: Past, present and future challenges. Ostrich 89: 139–143.

Martin RO, Senni C, D’Cruze NC. 2018. Trade in wild-sourced African grey parrots: Insights via social media. Global Ecology and Conservation 15: e00429.

Martin-Smith KM, Vincent ACJ. 2006. Exploitation and trade of Australian seahorses, pipehorses, sea dragons and pipefishes (Family Syngnathidae). Oryx 40: 141–151.

Moreau M-A, Coomes OT. 2007. Aquarium fish exploitation in western Amazonia: Conservation issues in Peru. Environmental Conservation 34: 12–22.

Ngo HN, Nguyen TQ, Phan TQ, van Schingen M, Ziegler T. 2019. A case study on trade in threatened tiger geckos (*Goniurosaurus*) in Vietnam including updated information on the abundance of the endangered *G. catbaensis*. Nature Conservation 33: 1–19.

Nijman V, Shepherd CR. 2010. The role of Asia in the global trade in CITES II-listed poison arrow frogs: Hopping from Kazakhstan to Lebanon to Thailand and beyond. Biodiversity and Conservation 19: 1963–1970.

Nijman V, Shepherd CR, Mumpuni, Sanders KL. 2012. Over-exploitation and illegal trade of reptiles in Indonesia. Herpetological Journal 22: 83–89.

Nijman V, Spaan D, Rode-Margono EJ, Wirdateti, Nekaris KAI. 2017. Changes in the primate trade in Indonesian wildlife markets over a 25-year period: Fewer apes and langurs, more macaques, and slow lorises. American Journal of Primatology 79: e22517.

Olden JD, Whattam E, Wood SA. 2021. Online auction marketplaces as a global pathway for aquatic invasive species. Hydrobiologia. Forthcoming.

Rabemananjara FCE, Rasoamampionona Raminosoa N, Ravoahangimalala Ramilijaona O, Andreone F, Bora P, Carpenter AI, Glaw F, Razafindrabe T, Vallan D, Vieites DR. 2008. Malagasy poison frogs in the pet trade: A survey of levels of exploitation of species in the genus Mantella. Amphibian and Reptile Conservation 5: 3–16.

Raghavan R, Ali A, Philip S, Dahanukar N. 2018. Effect of unmanaged harvests for the aquarium trade on the population status and dynamics of redline torpedo barb: A threatened aquatic flagship. Aquatic Conservation: Marine and Freshwater Ecosystems 28: 567–574.

Raghavan R, Dahanukar N, Tlusty MF, Rhyne AL, Krishna Kumar K, Molur S, Rosser AM. 2013. Uncovering an obscure trade: threatened freshwater fishes and the aquarium pet markets. Biological Conservation 164: 158–169.

Rhyne AL, Tlusty MF, Schofield PJ, Kaufman L, Morris Jr JA, Bruckner AW. 2012. Revealing the appetite of the marine aquarium fish trade: The volume and biodiversity of fish imported into the United States. PLoS ONE 7: e35808–e35808.

Robinson JE, Fraser IM, John FAV St., Randrianantoandro JC, Andriantsimanarilafy RR, Razafimanahaka JH, Griffiths RA, Roberts DL. 2018. Wildlife supply chains in Madagascar from local collection to global export. Biological Conservation 226: 144–152.

Robinson JE, St. John FAV, Griffiths RA, Roberts DL. 2015. Captive reptile mortality rates in the home and implications for the wildlife trade. PLoS ONE 10: e0141460.

Rubec PJ, Cruz FP. 2005, Monitoring the chain of custody to reduce delayed mortality of net‐caught fish in the aquarium trade. SPC Live Reef Fish Information Bulletin 13: 13– 23.

Scheffers BR, Oliveira BF, Lamb I, Edwards DP. 2019. Global wildlife trade across the tree of life. Science 366: 71–76.

Schmidt C, Kunzmann A. 2005. Post‐harvest mortality in the marine aquarium trade: A case study of an Indonesian export facility. SPC Live Reef Fish Information Bulletin 13: 3–12.

Shaw EM, Bennett SP, Wheater CP. 2011. Distribution of *Brachypelma vagans* (Theraphosidae) burrows and their characteristics in Belize over two years. The Journal of Arachnology 39: 515–518.

Shuman CS, Hodgson G, Ambrose RF. 2004. Managing the marine aquarium trade: Is eco-certification the answer? Environmental Conservation 31: 339–348.

Steinmetz M, Pütsch M, Bisschopinck T. 1998. Transport mortality during the import of wild caught birds and reptiles to Germany: An investigation (including a study on pre-export-conditions in the United Republic of Tanzania). German Federal Agency for Nature Conservation: Bonn, Germany.

Strecker AL, Campbell PM, Olden JD. 2011. The aquarium trade as an invasion pathway in the Pacific Northwest. Fisheries 36: 74–85.

Su S, Cassey P, Blackburn TM. 2014. Patterns of non-randomness in the composition and characteristics of the Taiwanese bird trade. Biological Invasions 16: 2563–2575.

Vagelli AA. 2004. Significant increase in survival of captive-bred juvenile Banggai Cardinalfish *Pterapogon kauderni* with an essential fatty acid-enriched diet. Journal of the World Aquaculture Society 35: 61–69.

Vall-llosera M, Cassey P. 2017. Physical attractiveness, constraints to the trade and handling requirements drive the variation in species availability in the Australian cagebird trade. Ecological Economics 131: 407–413.

Van Wilgen NJ, Wilson JRU, Elith J, Wintle BA, Richardson DM. 2010. Alien invaders and reptile traders: What drives the live animal trade in South Africa? Animal Conservation 13: 24–32.
