# Supplementary material for: The International Vertebrate Pet Trade Network and Insights from US Imports of Exotic Pets
Source: Bioscience. 2021 Jun 9;71(9):977–90. doi: 10.1093/biosci/biab056 (PMC8407969; doi:10.1093/biosci/biab056)
Supplement: biab056_Supplemental_Files [file biab056_supplemental_files.zip › Supplementary_Materials_S2.docx]

**Supplementary Materials S2** – LEMIS shipment records and exclusion of non-pet shipments

To analyze the US pet trade, we obtained a comprehensive record of legal US imports of over 230,000 shipments of wild-caught and captively-bred vertebrates entering the US between 1999 and 2013 from the US Fish and Wildlife Service Law Enforcement Management Information System (LEMIS; processing methods are detailed in Rhyne et al. 2012 and Eskew et al. 2020). LEMIS contains records of the number of individuals of all live vertebrates imported into the US at the taxonomic level of either genus or species. The inclusion within LEMIS of species that have no legal mandate for tracking, such as through the Convention on International Trade in Endangered Species of Wild Fauna and Flora (CITES), makes it one of the most comprehensive data sources on the wildlife trade. The LEMIS shipment records include the US port of entry, the country from which individuals were shipped, and the country from which they were sourced either from the wild or from captive breeding.

We divided shipment records within LEMIS into five broad animal clades – amphibians, birds, freshwater/marine fishes, mammals (excluding domesticated dogs, cats, and horses), and reptiles. We then further reduced this dataset to include only animals imported as pets. This assessment was based on the known use of each genus or species from information extracted from the shipment declaration forms within LEMIS (see fws.gov/le/declaration-form-3-177.html for details) and from the identity of the importing company if reported.

First, we excluded all records with no genus or species names because these animals could not be assessed for their relevance to the pet trade. These exclusions were particularly severe for the fish data (~83% of records removed), most of which were likely high-volume imports of personal or commercial food fish. Exclusions were comparatively less severe for the reptile records (~17% removed), and negligible for the amphibians (<1% removed), birds (<1%) and mammals (<1%). We also excluded shipments records that listed the US as the country of origin as these were likely animals initially harvested in the US, exported to another country for breeding or some other purpose, then re-exported back into the US (Eskew et al. 2020).

Next, we individually assessed all records for genera or species that constituted more than 0.01% of total animal clade trade volume across years. The countries and companies conducting these shipments were examined and all records for individual genera or species were excluded if the pet trade was generally not the primary reason for imports. Principal examples of exclusions include the *Rana* genus for the amphibians (usually bullfrogs imported for food and research), *Phasianus colchicus* for the birds (Common pheasant imported for food and game), *Lates calcarifer* for the fishes (Barramundi imported for food), *Bison bison* for the mammals (food, livestock, and zoos), and *Pelodiscus sinensis* for the reptiles (Chinese shoftshell turtle imported for food). Note that some individual shipments of excluded genus or species can occasionally be for sale as pets, but the vast majority of imports are for non-pet purposes.

Finally, all other imported genera or species that constituted less than 0.01% of total trade volume, which were usually small and infrequent imports with little to no detail on the companies involved, were assessed at the family level. These animals were excluded if they belonged to a family that was generally not imported for the pet trade, which we based on our previous assessments of individual genera and species imports greater than 0.01% of trade volume. For example, we determined that all individual, high volume (>0.01%) Iguanidae genus/species imports were for the pet trade, therefore we assumed that all low volume imports (<0.01%) of the Iguanidae family were also related to the pet trade. We also specifically excluded imports of *Canis lupus familiaris* (or *Canis familiaris*) and *Felis catus* as these are domesticated mammals.

The finalized dataset included 38,485 amphibian, 11,210 bird, 5,667 mammal, 38,974 freshwater/marine fish, and 89,720 reptile shipment records that together detailed the trade of over 187 million individual animals over 15 years. From each of these shipment records, we extracted information on the US port of import, scientific name of the imported pets, import quantity, and country of origin.

**References**

Eskew EA, White AM, Ross N, Smith KM, Smith KF, Rodríguez JP, Zambrana-Torrelio C, Karesh WB, Daszak P. 2020. United States wildlife and wildlife product imports from 2000–2014. Scientific Data 7: 22.

Rhyne AL, Tlusty MF, Schofield PJ, Kaufman L, Morris Jr JA, Bruckner AW. 2012. Revealing the appetite of the marine aquarium fish trade: The volume and biodiversity of fish imported into the United States. PLoS ONE 7: e35808–e35808.

Romagosa C. 2014. Patterns of live vertebrate importation into the United States: Analysis of an invasion pathway. Pages 115–146 in Keller RP, Cadotte MW, and Sandiford G, eds. Invasive Species in a Globalized World: Ecological, Social, and Legal Perspectives on Policy. Chicago University Press.
