# Supplementary material for: The International Vertebrate Pet Trade Network and Insights from US Imports of Exotic Pets
Source: Bioscience. 2021 Jun 9;71(9):977–90. doi: 10.1093/biosci/biab056 (PMC8407969; doi:10.1093/biosci/biab056)
Supplement: biab056_Supplemental_Files [file biab056_supplemental_files.zip › Supplementary_Materials_S3.docx]

**Supplementary Materials S3** – US case study methods and supplementary results

To quantify spatial patterns in the volume and diversity of pet exports and imports to the US, we mapped the quantity (all genus and species records; figures S3.1 and S3.2) and species richness (species records only) of pets exported from each country (figure 2) and imported to each US port of entry (figure 3) averaged from 1999 through 2013. Total export and import quantity and richness values were first calculated across all shipment records within each year, then averaged across years for each country or port and each animal clade (the top five are listed in table 1).

Next, we compared the number of species exported from a given country (termed ‘export richness’) to how many traded species naturally occur within that country (termed ‘native richness’; figure 2). To perform this analysis, we compared export species richness averaged across years for each country to the total number of amphibian, bird, fish, mammal, and reptile species found in the LEMIS database native to that country. We made native species richness distribution maps in two steps: (1) matching and resolving the individual species names for each animal clade in our LEMIS database to an associated species name in a relevant distribution database; and (2) counting the number of species distributions that overlapped each country worldwide. We matched the species names recorded in LEMIS with spatial distributions datasets for amphibians (IUCN 2018), birds (BirdLife International 2017), mammals (IUCN 2018), and reptiles (Roll et al. 2017). Comprehensive country-level species lists are not readily available for freshwater fishes, so we used watershed-based freshwater fish distribution data from Tedesco et al. (2017) to create a global native richness map of traded freshwater fishes by watershed rather than countries. We excluded marine fishes from this analysis because marine distributions cannot be directly overlaid on country export values.

Three matching techniques were used to associate the LEMIS species names with their respective distribution datasets. First, we directly matched LEMIS species names to the species name in the distribution database. Next, we took the remaining unmatched names and resolved them using three taxonomic databases (Catalogue of Life, Global Biodiversity Information Facility, and the National Center for Biotechnology Information) using the R package ‘taxize’ (Chamberlain et al. 2019). Any remaining unmatched species we then assessed individually. A total of 2387 species were successfully matched while 130 species had no data available in their respective distribution datasets (see table S3.1 for breakdown by animal clade).

To create the native species richness maps by country, we extracted the distribution polygons for each matched species from their respective distribution datasets and ‘dissolved’ these polygons such that each species was represented by one distribution polygon (which could be non-continuous in space, i.e., a multipart polygon). Native species richness was then calculated by country for each animal clade by summing the total number of unique species distribution polygons that intersected the borders of each country. Spatial selections and dissolves were performed in QGIS (QGIS Development Team 2020) and all other analyses were performed in R (R Core Team 2018) using the packages ‘raster’ (Hijmans 2020), ‘sp’ (Bivand et al. 2013), and ‘rgeos’ (Bivand and Rundel 2019). Links to online, interactive versions of our export quantity/richness and native distribution maps (see Fig. 3 and Fig. S3.2) are provided in Table S3.2.

***Network analyses***

To quantify spatial and temporal patterns in network connectivity between US ports of import and export countries (figures 4 and 5), we calculated two metrics commonly used for directed graphs (i.e., networks with directional links): (i) the number of incoming trade connections to each US port, which represents the number of countries from which each port receives imports; and (ii) the number of outgoing trade connections from each exporting country, which represents the number of different US ports with which each country trades. The number of incoming and outgoing trade connections provides simple, spatially explicit measures of pet transport, such as which US ports tend to consolidate pets from the highest number of source regions (i.e., more incoming connections) and which exporting countries tend to disperse their pets across multiple US ports of entry (i.e., more outgoing connections).

For each animal clade, we averaged the number of incoming trade connections for each US port, or the number of outgoing trade connections for each exporting country, across years. We then plotted the frequency distributions of these averaged incoming or outgoing connections using histograms as a visual aid to compare values among animal clades (figure 4 and figure S3.3).

Finally, we used Analysis of Covariance (ANCOVA) models to quantify how these incoming and outgoing trade connections have changed over time (figure 5). These models used a continuous response variable of the average number of incoming connections within each year (averaged across all importing ports), or the average number of outgoing connections within each year (averaged across all exporting countries), for each animal clade. Model predictor variables included a continuous variable for each year (1999–2013), a categorical variable for each animal clade, and an interaction term between the two variables to determine how incoming and outgoing connections have changed over time across animal clades. Statistical significance of main effects was assessed at the *P* < 0.05 level. Average incoming and outgoing trade connections were significantly different among animal clades and changed over time (evidenced by significant Year:Clade terms; Incoming: *n*=75, F_4,65_=20.0, R^2^=0.91, *P*<0.001; Outgoing: *n*=75, F_4,65_=2.7, R^2^=0.84, *P*=0.038). Both the average number of incoming trade connections to US ports and outgoing trade connections from exporting countries tended to decrease from 1999 to 2013 for amphibians (Incoming: -3.6; Outgoing: -0.2) and reptiles (Incoming: -3.8; Outgoing: -0.3), and tended to increase for fishes (Incoming: +1.8; Outgoing: +0.4) and mammals (Incoming: +0.3; Outgoing: +0.5) (based on predicted linear relationships for each clade). Conversely, for birds, the average number of incoming connections to US ports tended to decrease over time (-1.9), whereas the average number of outgoing connections from exporting countries slightly increased (+0.1).

**References**

BirdLife International. 2017. IUCN Red List for birds. BirdLife International. (27 July 2018; *www.birdlife.org*)

Bivand RS and Rundel C .2019. rgeos: Interface to Geometry Engine – Open Source ('GEOS'), v0.5-2. (6 January 2021; *https://CRAN.R-project.org/package=rgeos*)

Bivand RS, Pebesma E, Gomez-Rubio V. 2013. Applied spatial data analysis with R, second edition. Springer.

Chamberlain S, et al. 2019. taxize: Taxonomic information from around the web, v0.9.5. (6 January 2021; *https://github.com/ropensci/taxize*)

Henry L and Wickham H. 2019. purrr: Functional programming tools, v0.3.0. (6 January 2021; *https://CRAN.R-project.org/package=purrr*)

Hijmans RJ. 2020. raster: Geographic data analysis and modeling, v 3.1-5. (6 January 2021; *https://CRAN.R-project.org/package=raster*)

[IUCN] International Union for Conservation of Nature. 2018. The IUCN Red List of Threatened Species, Version 6.1. IUCN. (11 February 2019; *www.iucnredlist.org*)

Müller K Wickham H. 2019. tibble: Simple data frames, v 2.0.1. (6 January 2021; *https://CRAN.R-project.org/package=tibble*)

QGIS Development Team. 2020. QGIS v3.12.1 Geographic Information System. Open Source Geospatial Foundation Project. (31 March 2020; *http://qgis.osgeo.org*)

R Core Team. 2018. R: A language and environment for statistical computing. R Foundation for Statistical Computing, Vienna, Austria. (6 January 2021; *www.R-project.org*)

Roll U, et al. 2017. The global distribution of tetrapods reveals a need for targeted reptile conservation. Nature Ecology & Evolution 1: 1677–1682.

Sumner MD. 2019. spdplyr: Data manipulation verbs for the spatial classes, v0.3.0. (6 January 2021; *https://CRAN.R-project.org/package=spdplyr*)

Tedesco PA, et al. 2017. A global database on freshwater fish species occurrence in drainage basins. Scientific Data 4: 170141.

Wickham H. 2016. ggplot2: Elegant graphics for data analysis. Springer-Verlag.

Wickham H. 2019. stringr: Simple, consistent wrappers for common string operations, v1.4.0. (6 January 2021; *https://CRAN.R-project.org/package=stringr*)

Wickham H, François R, Henry L and Müller K. 2018. dplyr: A grammar of data manipulation, v0.7.8. (6 January 2021; *https://CRAN.R-project.org/package=dplyr*)

Wickham H and Ruiz E. 2019. dbplyr: A 'dplyr' back end for databases, v1.4.2. (6 January 2021; *https://CRAN.R-project.org/package=dbplyr*)

***Table S3.1. The total number of unique pet species for each animal clade in the LEMIS records compared to how many of these species had available data in their respective distribution datasets.***

| **Animal clade** | **Number of LEMIS pet species** | **Number of species successfully matched** | **Distribution database** |
| --- | --- | --- | --- |
| Amphibians | 342 | 307 | IUCN 2018 |
| Birds | 680 | 650 | BirdLife International 2017 |
| Freshwater fishes | 174 | 152 | Tedesco et al. 2017 |
| Mammals | 145 | 135 | IUCN 2018 |
| Reptiles | 1149 | 1143 | Roll et al. 2017 |

***Table S3.2. Links to interactive maps showing our data on country-level export quantity (‘Exp. vol.’), export richness (‘Exp. rich.’), and native richness (‘Nat. rich.’) for each animal clade. The amphibian, bird, mammal, and reptile maps were rendered with the ‘qgis2web’ plugin in QGIS (QGIS Development Team 2020) using Leaflet, whereas the map for fishes used OpenLayers owing to problems with showing both basin- and country-level data in Leaflet. Note: Leaflet displays all export quantities regardless of magnitude so a slider has been included to enable the removal of low quantity values in these maps.***

| **Animal clade** | **Map type** | **Link** |
| --- | --- | --- |
| Amphibians | Leaflet | https://jamessinclair270.github.io/LEMIS-amphibian-maps/ |
| Birds | Leaflet | https://jamessinclair270.github.io/LEMIS-bird-maps/ |
| Freshwater fishes | OpenLayers | https://jamessinclair270.github.io/LEMIS-fish-maps/ |
| Mammals | Leaflet | https://jamessinclair270.github.io/LEMIS-mammal-maps/ |
| Reptiles | Leaflet | https://jamessinclair270.github.io/LEMIS-reptile-maps/ |

**References**

QGIS Development Team. 2020. QGIS v3.12.1 Geographic Information System. Open Source Geospatial Foundation Project. (31 March 2020; *http://qgis.osgeo.org*)


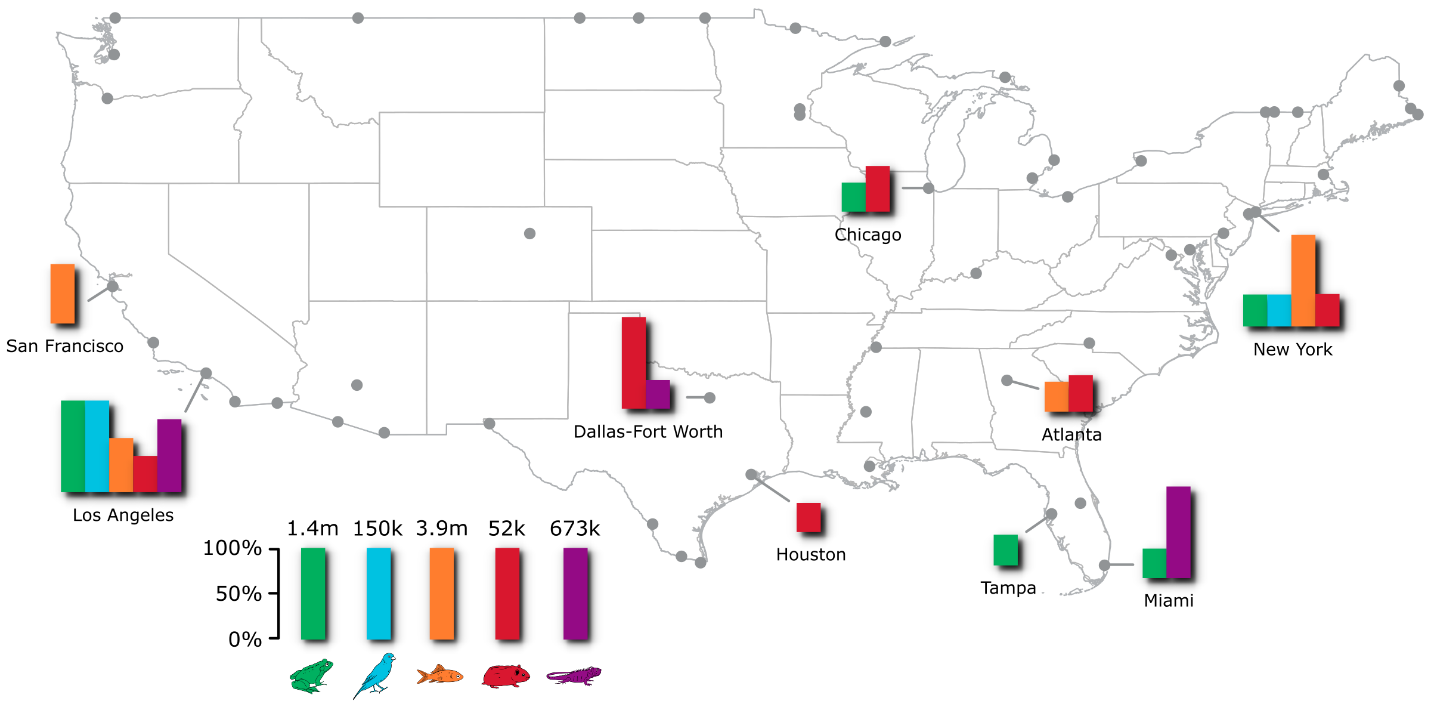


***Figure S3.1. Average quantity of amphibians (green), birds (blue), fishes (orange), mammals (red), and reptiles (purple) imported to each US port of entry for sale as pets from 1999 through 2013. There are strong spatial patterns in where different animal clades tend to be imported into the US such that only a handful of ports – primarily Dallas-Fort Worth, Los Angeles, Miami, and New York – receive the majority of imports. Groups of bars are geographically positioned near their respective ports and individual bar heights are scaled relative to the maximum import quantity for each animal clade (listed at the top of each bar in the legend). Ports whose imports constitute less than 5% of total import quantity are plotted as grey points.***


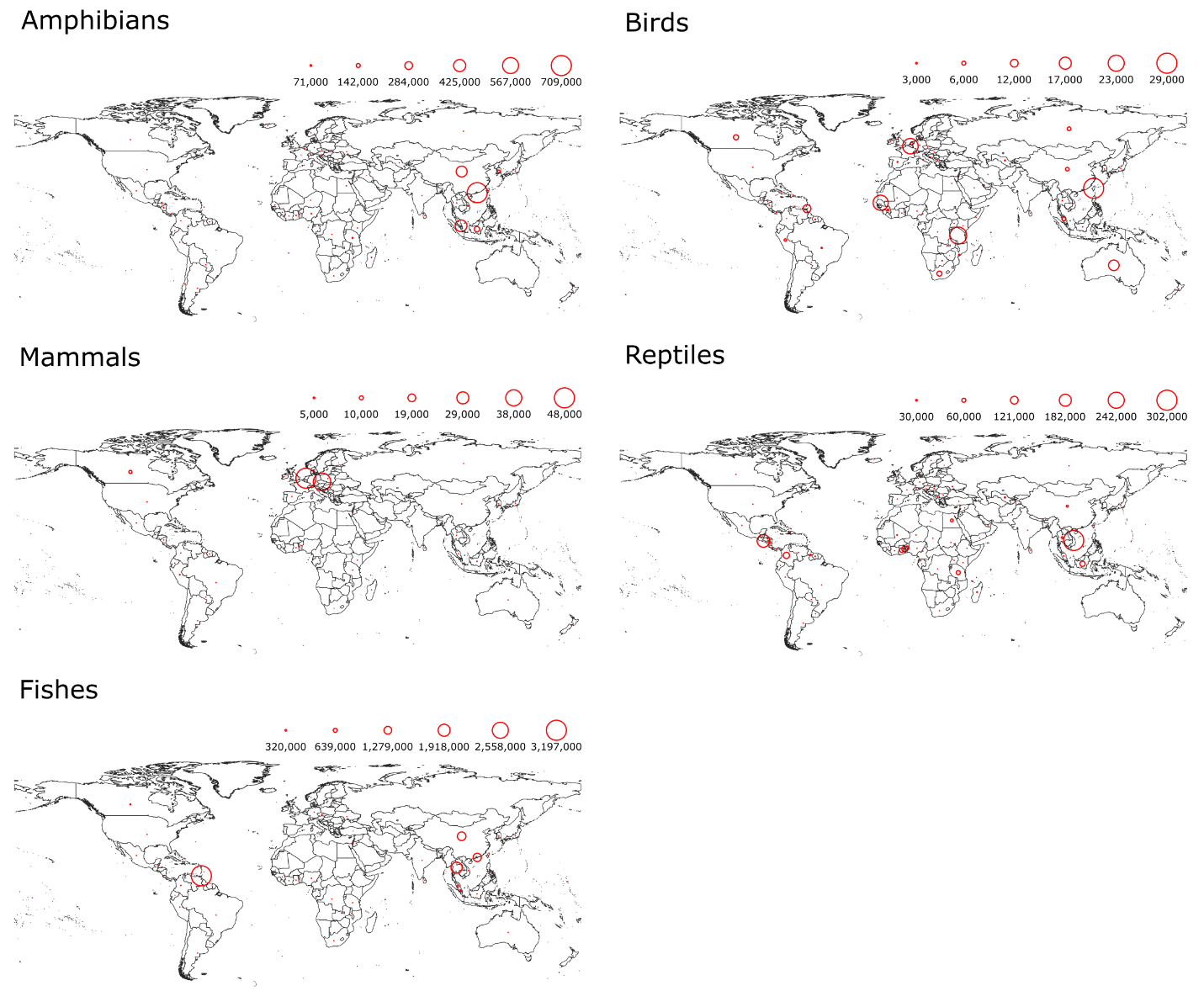


***Figure S3.2. Average quantity of amphibians, birds, fishes, mammals, and reptiles exported from each country to the US for sale as pets from 1999 through 2013. Red circles indicate export country node locations and are sized relative to their maximum values (rounded to the nearest 1000).***


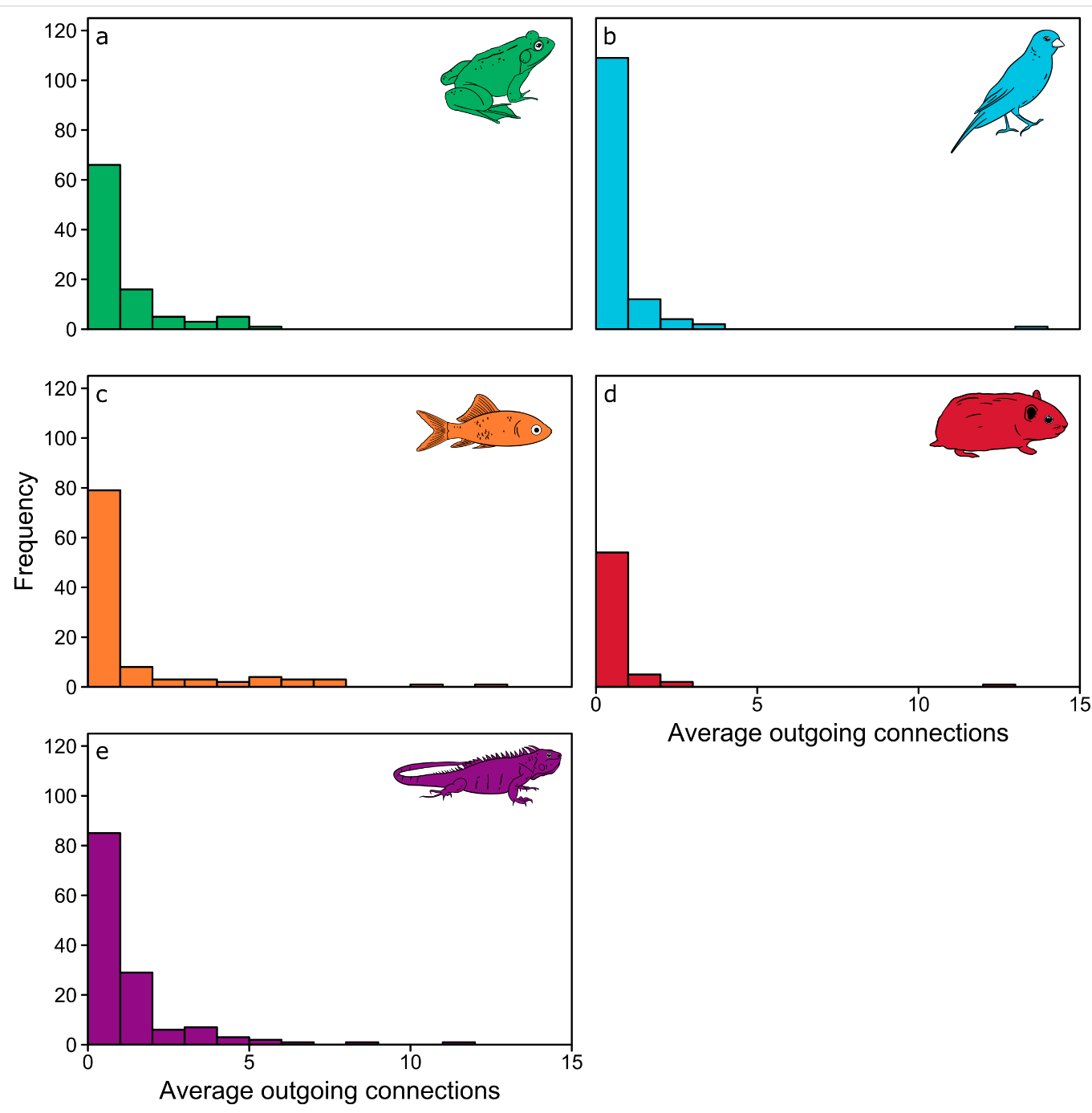


***Figure S3.3. Frequency distributions of outgoing trade connections for pet (a) amphibians, (b) birds, (c) fishes, (d) mammals, and (e) reptiles exported from each country to the US. The outgoing connections for each country are calculated as the number of different US ports to which each country exports for each animal clade averaged from 1999 through 2013. These right-skewed distributions indicate that most countries are exporting to a single US port, whereas a small number of countries (e.g., Canada) are exporting animals to many different ports.***
